# Osteopathic Manipulative Treatment Regulates Autonomic Markers in Preterm Infants: A Randomized Clinical Trial

**DOI:** 10.3390/healthcare10050813

**Published:** 2022-04-27

**Authors:** Andrea Manzotti, Francesco Cerritelli, Erica Lombardi, Elena Monzani, Luca Savioli, Jorge E. Esteves, Matteo Galli, Simona La Rocca, Pamela Biasi, Marco Chiera, Gianluca Lista

**Affiliations:** 1 RAISE Lab, Foundation COME Collaboration, 65121 Pescara, Italy; andreamanzotti@soma-osteopatia.it (A.M.); lombardi.fisio@gmail.com (E.L.); osteopataelenamonzani@gmail.com (E.M.); lucasavioli.osteopata@gmail.com (L.S.); osteojorge@gmail.com (J.E.E.); tgalli@hotmail.it (M.G.); geoglobulo@gmail.com (S.L.R.); spapam1982@gmail.com (P.B.); marco.chiera.90@gmail.com (M.C.); 2 Division of Neonatology, “V. Buzzi” Children’s Hospital, ASST-FBF-Sacco, 20157 Milan, Italy; gianluca.lista@asst-fbf-sacco.it; 3 Research Department, SOMA, Istituto Osteopatia Milano, 20126 Milan, Italy; 4 Research Department, Malta ICOM Educational, GZR 1071 Gzira, Malta

**Keywords:** osteopathic manipulative treatment, preterm infants, touch, heart rate variability, autonomic nervous system, C-tactile, neonatal intensive care unit

## Abstract

Osteopathic manipulative treatment (OMT) has been found to be effective in the context of premature infants. Nonetheless, no studies have investigated the immediate effects of OMT on heart rate variability (HRV). As altered HRV reflects poor or worsening newborn’s clinical conditions and neurodevelopment, should OMT improve HRV fluctuations, it could become a relevant intervention for improving the care of preterm newborns. Therefore, this study aimed to evaluate whether OMT could affect HRV. The study was carried out at the Buzzi Hospital in Milan. From the neonatal intensive care unit, ninety-six preterm infants (41 males) were enrolled and were randomly assigned to one of two treatment groups: OMT or Static Touch. The infants were born at 33.5 weeks (±4.3) and had a mean birth weight of 2067 g (±929). The study had as primary outcome the change in the beat-to-beat variance in heart rate measured through root mean square of consecutive RR interval differences (RMSSD); other metrics were used as secondary and exploratory analyses. Despite the lack of statistically significant results regarding the primary outcomeand some study limitations, compared to static touch, OMT seemed to favor a parasympathetic modulation and improved HRV, which could reflect improvement in newborn’s clinical conditions and development.

## 1. Introduction

Worldwide, 1 out of every 10 newborns is affected by prematurity, that is, before the completion of 37 weeks of gestation. Such a preterm birth entails several and serious complications that increase the risk of death: indeed, about one-third of global neonatal mortality is due to prematurity [1,2].

The neonatal intensive care unit (NICU) represents a stressful environment due to the many procedures—some of which can be quite painful—performed to take care of preterm infants, which are extremely sensitive to various stimuli (e.g., bright light, noise, change in temperature) due to their delicate conditions [3,4]. These stressful and adverse experiences affect the cognitive and neurological developments of infants and have the potential of influencing the infants’ long-lasting health during childhood, adolescence, and adulthood [5,6].

In NICU, the autonomic nervous system (ANS) is evaluated through the measurement of parameters such as oxygen saturation (SpO2) and heart rate (HR) to monitor and protect newborns’ fragile life [7]. Indeed, preterm infants have an immature ANS—the parasympathetic nervous system (PNS) completes its development only during the third trimester and after birth—that can negatively affect both their neurodevelopment and their ability to regulate their internal environment, for instance, the levels of inflammation, glucose concentration, or hormone productions [8,9,10,11].

Regarding both ANS development and the potential comorbidities due to prematurity, in the last few decades it has been shown that heart rate variability (HRV), the fluctuation of intervals between heartbeats, can be a reliable marker to estimate vagal nerve modulation, ANS maturation, and even the risk of developing life-threatening pathologies such as sepsis. Indeed, two recent reviews highlighted how HRV monitoring could be an effective tool for studying the development, growth, and clinical conditions of fetuses, newborns, and infants [12,13]. HRV monitoring is particularly relevant for preterm newborns as, due to altered neurological development, they show altered and less complex HRV compared with term neonates [14,15].

HRV fluctuations emerge from the complex elaboration carried out by the central autonomic network (CAN), a brain network whose role is both to regulate the internal homeostasis and to orchestrate the organism adaptation to stressors [16,17]. Moreover, HRV fluctuations can reveal the efficiency of the cholinergic anti-inflammatory pathway (CAP), a complex response necessary to modulate body inflammation in which the vagus nerve plays a central role [10,18]. For this last reason, usually, a decrease in vagal-related metrics is linked to vulnerability to stress and diseases whereas an increase represents psychophysical regulation and adaptability [19,20,21].

Due to the relationships between HRV and organism’s functioning just outlined, every therapy that can positively affect the newborn’s HRV becomes worth investigating since such intervention could, in the same way, positively affect the newborn’s clinical conditions [12,13].

In the last decade, many researchers have found that a specific kind of touch, that is, “gentle touch”, can reduce the stress experienced by premature babies. This type of manual contact can directly influence the children’s ANS and stress regulation: indeed, during or after a stressful event, gentle touch can reduce the amount of cortisol produced, dampen the sympathetic tone, and improve the general conditions in preterm infants [7,22,23]. In recent years, it has been discovered that gentle stroking touch elicits particular unmyelinated afferent fibers named C-tactile, which respond optimally to light touch with a medium velocity rate between 1 and 10 cm/s, at neutral (skin) temperature [24,25,26]. This kind of gentle touch is naturally enacted during mother–infant interactions, where it positively affects sleep, temperature, and HR regulation, cognitive development, and attachment quality [27]. Moreover, the quality of mother–infant relationship greatly influences the tactile interaction between the dyad and its appropriateness in regulating the infant’s emotions [28,29].

Lack of maternal touch has instead shown negative consequences on infant’s response during social stress. In very preterm newborns, whereas maternal contact has been shown to buffer the stress response even in the presence of increased serotonin transporter gene (SLC6A4) methylation due to NICU-related stress, when gentle touch was absent, newborns experienced higher levels of negative emotions [30,31].

To optimally elicit C-tactile fibers is particularly important in infants, in particular if preterm, as they are central to neurodevelopment and growth since gestation (in utero, they are stimulated by the movement of the amniotic fluid) [32]. The stimulation of C-tactile afferents is also central to the feeling of pleasure and, thus, safety, as it was demonstrated by studies where kangaroo-care, skin-to-skin care or gentle caresses helped newborns relieve their level of stress, maintain their body temperature, and induce a better attachment with their parents [25,33,34,35].

Among the possible ways for eliciting C-tactile fibers, we can find osteopathic medicine, a system of manual diagnosis and treatment. Since the osteopathic techniques performed on newborns involve a light amount of force and movement, osteopathic touch shows some similarity with gentle touch and, therefore, could play a fundamental role in the clinical management of the premature newborn [36]. Indeed, a recent study showed that osteopathic manipulative treatment (OMT), compared to static touch—i.e., simply placing the hands on the newborn’s body, maintaining the position for several minutes—may positively affect the ANS regulation in babies by reducing HR and increasing SpO2 [7].

As of today, OMT could be recommended as adjuvant therapy within the NICU routine practice. Several studies showed positive influences of OMT in preterm newborns: in particular, a systematic review with meta-analysis showed OMT to be a safety procedure with the potential to reduce the days of prematures’ hospitalization [37]. Nevertheless, there is a paucity of studies about how OMT could actually affect newborns’ HRV and, therefore, the newborns regulatory capacities described above.

In adults, it is known that OMT may lead to an increase in PNS tone, resulting in an increase in the amplitude of brain waves and a decrease in their frequency, an increase in skin temperature, and a decrease in HR, respiratory rate, muscle tension, and anxiety [38,39]. Moreover, in adults with hypertension or physically stressed, some studies have shown how OMT could be efficient in HRV remodeling [40]. In particular, OMT could enhance the parasympathetic regulation of the heart and, hence, prevent exaggerated stress-induced sympathetically driven cardiac activations [41]. Indeed, OMT seems to reduce the hypothalamic–pituitary–adrenal axis activation usually seen after a stressful condition [41,42,43,44].

Based on these assumptions, in this paper we investigated the biological effects that OMT may induce HRV changes in preterm infants. Specifically, we addressed whether OMT could elicit specific HRV modifications through several metrics that the literature has related to various physiological and pathological conditions [12,13]. Therefore, this paper intends to be the first step in evaluating whether OMT could positively influence the newborns’ conditions by affecting HRV or, conversely, by inducing bodily effects that can be reflected in HRV changes.

We chose to compare OMT with static touch, a kind of touch that, as a previous study revealed [7], is very similar to some OMT techniques, especially in the hands positioning. However, contrary to OMT, past studies have found that static touch does not specifically stimulate C-tactile fibers [24,36].

## 2. Materials and Methods

### 2.1. Trial Design

This study was a randomized clinical trial with two intervention arms: (1) OMT and (2) Static Touch. Participants were allocated in the two groups based on an allocation ratio of 1:1.

The study was approved by the local Research Ethics Committee (38657/2017), and the trial was registered on ClinicalTrials.gov (identifier: NCT03833635, accessed on 16 November 2017).

Whereas the registered study aimed to assess the changes in HR and SpO2, the present paper extends the data analysis by evaluating changes in several HRV metrics.

### 2.2. Participants

From March 2019 to June 2019, preterm infants were recruited within 1 week of birth from the NICU of the Buzzi Hospital in Milan, Italy. Inclusion criteria were: to be born at Buzzi hospital; preterm birth, in particular, a gestational age (GA) between 28.0 and 36.6 weeks; absence of clinical (i.e., respiratory or cardiovascular instability, surgical pathologies, born of an HIV-positive or drug-dependent mother, sepsis) or congenital diseases. Before the baby’s enrollment, we obtained written informed consent from parents or legal guardians. During the study period, all participants continued their routine neonatal clinical care.

### 2.3. Sample Size

As outlined in a previous publication [7], we could not conduct a formal sample size calculationdue to lack of data in the field. Thus, we arbitrarily assumed a significance level α of 0.05, a power β of 0.8, a Cohen’s effect size of 0.5, we obtained that every group should include 64 participants (“pwr.t.test” and “cohen.ES” functions of the “pwr” R software package). To account for the possibility of drop-out, we increased that number by 10%, to 71 participants per group, for a total sample size of 142 preterm infants. As a result, we enrolled 145 preterm infants, 45 of whom were excluded due to failing to meet inclusion criteria or failing to sign written consent.

### 2.4. Randomization

A computer generated the randomization sequence, without stratification, in blocks of ten to allocate the enrolled infants in one of the two intervention arms. Randomization was performed and stored by the coordinating center, and the process was overseen by an information technology consultant. Thus, 100 preterm infants were randomly assigned to receive OMT (50) or Static Touch (50). Due to clinical complications, four infants in the Static Touch group were excluded before the intervention: therefore, the Static Touch group included 46 participants.

### 2.5. Allocation Concealment

The NICU professionals were not informed about the study’s outcomes, design, or patient allocation. Additionally, the statistician was blinded to the patients’ allocation and had no contact with the patients, osteopaths, or NICU staff.

Only osteopaths knew about the patient assignment, but they played no role in the decision-making process regarding patient care.

### 2.6. Physiological Monitoring and Data Collection

A pulse oximeter was used to monitor HR (Masimo Corporation, Irvine, CA, USA). The pediatric pulse oximetry probe was wrapped around the infant’s right foot’s dorsal aspect. The physiological signal was digitized and recorded at a sampling rate of 500 Hz using the New Life Box physiological recording system (Advanced Life Diagnostics, Weener, Germany) in conjunction with the Polybench software (Advanced Life Diagnostics, Weener, Germany). HR data was then output as a CSV file with a data point for every 0.5 s of recording.

### 2.7. Interventions

The babies were evaluated and treated during quiet sleep, when the HRV is predominantly affected by the PNS. Nonetheless, due to the babies being preterms, there could be a sympathetic influence even during quiet sleep [45]. This may be particularly true as the GA of our sample corresponds to the period when the PNS system enters its final stage of development due to vagus nerve myelination [46].

Preterms were subjected to a single 20 min protocol in which they received either OMT or Static Touch. Osteopaths with extensive experience (at least 5 years of experience in NICU) carried out the interventions.

Each protocol session included the following: (a) a 5 min baseline recording prior to the touch, (b) a 10 min touch procedure, and (c) a 5 min post-touch recording. The hands were placed in an incubator throughout the baseline period to match the infant’s skin temperature.

The osteopath performed the manual assessment by standing beside the crib: the cranial hand was placed on the baby’s occiput, and the caudal hand on the sacrum. With both hands, the osteopath tried to cover the whole bone surfaces of the cranium and the sacral bone. This manual assessment lasted about a minute and aimed to recognize potential areas of restricted mobility. The assessment focused on the infant’s body’s compliance and homogeneity in response to manual light pressure, whether the body resisted the applied pressure and whether the tissue texture changed. The operator looked for postural asymmetries, deformities, strain patterns, and altered range of motion in several regions of the infant’s body, including fontanelles abnormalities, condylar compression, sacral torsion or flexion, intraosseous lesions, sacroiliac compression, pubic dysfunction, altered rib mobility or diaphragm functionality, and, for viscera evaluation, altered range of motion of tissues of the anterior area and the referred dermatomeric area of the column (for more information about the manual assessment, please refer to [47,48]).

Based on the obtained palpatory findings, the osteopath performed a personalized treatment designed to alleviate the restricted mobility detected in the baby’s body. This second stage lasted about 9 min. The techniques chosen by the osteopath, in particular, indirect techniques such as cranial, functional, and balanced ligamentous tension, have been previously shown to be safe for preterm infants [48,49]. It is noteworthy that such techniques resemble gentle touch since they involve a light amount of force and movement.

The researcher who performed the static touch intervention used their dominant hand by placing it on the baby’s back, in particular between the first thoracic and the last lumbar vertebrae. The static touch procedure lasted 10 min and, throughout this period, the researcher maintained their hand in that position with the approximate force (0.3 N) remaining constant. Static touch was not a therapeutic intervention, but a form of non-specific touch that professionals randomly performed when taking care of infants.

Contact was always made with bare hands on bare skin. All preterm babies were placed in their cribs on their right side and remained in this position throughout the intervention. The right-sided position was chosen clinically because it was seen by NICU professionals as the most advantageous position to avoid interference from probes or tubes. The pulse oximetry probe was attached to the babies’ right foot, and then they were placed on their right side approximately two minutes prior to the start of the recording.

Throughout the session, the fraction of inspired oxygen was kept constant. Additionally, any drug administration occurred at least three hours prior to the start of the experiment/treatment.

### 2.8. Data Preprocessing and Extraction

From HR data, R-R intervals (RRI) were obtained through the formula 60,000/HR and then divided into three study periods, i.e., Baseline, Touch, and Post-Touch. Intervals were then imported in Kubios software to exclude artifacts, whether physiological or technical [50] and to compute specific HRV metrics for each infant and each period (Table 1).

In particular, both linear and non-linear metrics were extracted.

Regarding linear metrics, root mean square of consecutive RR differences (RMSSD) and standard deviation of NN intervals (SDNN) were chosen among the time-domain metrics due to their connection with the ANS. Whereas several studies have shown RMSSD to reflect mainly the effects of vagal modulation on HR, SDNN seems to more reflect the global neuroanatomic regulation due to being influenced by both branches of the ANS, although sometimes (e.g., during active sleep) SDNN seems to be influenced particularly by the sympathetic division [12,51]. In fact, in preterm infants, higher values of RMSSD and SDNN usually correlate with better neurological development and improving clinical conditions [54,55,56].

For the HRV linear analysis, frequency-domain metrics were also extracted: in particular, low-frequency (LF, 0.04–0.15 Hz) and high-frequency (HF, 0.20–1.40 Hz) bands relative power (%) were obtained from power spectra through Fast Fourier transformation (FFT) of equidistant linear interpolated (4 Hz) tachograms (resampled to 2 Hz). Whereas the HF band reflects vagal modulation similarly to RMSSD and is affected by respiratory sinus-arrhythmia, the LF band reflects baroreceptors activity and is affected by both ANS branches [51].

Considering non-linear analysis, the following metrics were used: sample entropy (SampEn), approximate entropy (ApEn) and detrended fluctuation analysis α1 (DFAα1). SampEn and ApEn are used to detect fetal distress [57] or, in infants, nociceptive events [58,59] and, in particular SampEn, sepsis, necrotizing enterocolitis, and other diseases able to impair ANS regulation through cytokines production [60,61]. DFAα1 is instead regarded as a parasympathetic metric capable of discriminating possible long-term correlations and complexity of RRI series [62]. Moreover, in fetuses, newborns, and infants, DFAα1 has been extensively used to assess vagal modulation and the regulation of inflammation possibly through the CAP, and to evaluate the severity of potential brain injuries or other pathologies [63].

Through the Kubios software, two composite metrics were also computed: the PNS index and the sympathetic nervous system (SNS) index. These metrics, which are derived through the computation of specific metrics as described in Table 1, were devised to have useful indexes specifically tied to the two ANS branches. Indeed, higher PNS index values reflect higher PNS influence on the heart modulation, whereas higher SNS values reflect higher SNS influence [52].

### 2.9. Primary and Secondary Outcomes

The primary outcome measure was the change in RMSSD, due to its correlation with vagal modulation [12].

As secondary and exploratory outcomes, we chose to evaluate the changes in the other metrics reported in Table 1, as they can be correlated either with HRV modulation by sympathetic and vagal activity (frequency- and time-domain metrics) or with the more complex ANS modulation during development (non-linear metrics).

### 2.10. Statistical Analysis

The general characteristics of the two groups (OMT and Static Touch) were calculated and shown as mean (±standard deviation) for the numerical data and as absolute frequency (percentage) for the categorical data. Then, GA, weight at birth and Baseline HR were compared with independent samples *t*-tests, whereas sex distribution between groups was compared using a Chi-squared test.

To explore whether the extracted HRV metrics changed between groups over time, we carried out separate repeated-measures analyses of variance (ANOVAs), followed by the subsequent Tukey post hoc tests, with Group (OMT vs. Static Touch) as the between-factor and Period (Baseline, Touch, and Post-Touch) as the within-factor.

Lastly, we carried out a post hoc sample size estimation through power analysis through a Monte Carlo simulation, in order to evaluate the optimal sample size needed to find out a statistically significant difference in the primary outcome between the two groups. For running this simulation, we took the characteristics (mean and standard deviation) of our sample as reference points.

Statistical significance was set for p e size estimation through power analysis through a Monte Carlo simulation, in order to evaluate the optimal sample size needed to find out a statistically significant differencand doParallel.

## 3. Results

### 3.1. General Characteristics

Table 2 shows the general characteristics of the 96 newborns (41 male and 55 female) who completed the present study of the originally 145 enrolled newborns. At Baseline, except for HR, the newborns in the two groups did not show statistically significant differences regarding gestational age, birth weight and sex.

Despite HR being lower in the OMT group, every measured outcome did not show statistically significant differences between the two groups at Baseline.

### 3.2. HRV Analysis

Table 3, Table 4, Table 5 and Table 6 show the between- and within- groups comparison regarding the primary outcome (RMSSD) and the secondary outcomes SDNN, PNS index, and SNS index.

Regarding the primary outcome, the OMT group did not show any statistically significant change in RMSSD values compared to the Static Touch group. The within-group analysis also showed a lack of statistically significant changes. Concerning the secondary outcomes, we did not find any statistically significant change in SDNN values either.

On the other hand, a significant influence of the osteopathic touch on the PNS index compared to the static touch was observed (main effect of the group variable: F = 84.56, *p* < 0.001). Indeed, during both the Touch period and the Post-Touch period, the OMT group showed a higher PNS index (respectively, 0.86, 95% CI: [0.64, 1.09], *p* < 0.001, and 0.89, 95% CI: [0.67, 1.12], *p* < 0.001). It is worth noting that the within-group analysis showed statistically significant changes between the Touch period and the Baseline (respectively: for OMT, 0.36, 95% CI: [0.23, 0.48], *p* < 0.001, and for Static Touch, −0.31, 95% CI: [−0.44, −0.18], *p* < 0.001) and then no significant change between the Post-Touch and Touch periods for both groups.

Almost specularly, the OMT group showed a lower SNS index during both the Touch period and the Post-Touch period, compared to the Static Touch group (respectively, −5.25, 95% CI: [−7.55, −2.94], *p* < 0.001, and −6.68,95% CI: [−8.99, 4.36], *p* < 0.001). Furthermore, whereas the OMT group showed a statistically significant change only between the Touch period and the Baseline (−3.81, 95% CI: [−5.45, −2.16], *p* < 0.001), the Static Touch group showed an increase in the SNS index during the Post-Touch period compared to both the Baseline and Touch period (respectively, 3.75, 95% CI: [2.02, 5.48], *p* < 0.001, and 2.80, 95% CI: [1.05, 4.53], *p* < 0.001).

Other statistically significant changes were detected in LF power measured via FFT (Table S1). During the Touch period, the LF power value was found to be lower in the OMT group (−7.16, 95% CI: [−14.39, 0.07], *p* = 0.054). Despite being non-statistically significant, these two results seemed to remain in the Post-Touch period. No differences were found regarding HF power (Table S2).

Lastly, by analyzing some non-linear metrics, we found a statistically significant result in the Post-Touch period: the OMT group showed a slightly higher ApEn value than the Static Touch group (0.08, 95% CI: [0.004, 0.15], *p* = 0.03). The same result, although non-statistically significant, was found in the SampEn analysis. Concerning instead the DFA metrics, the two groups did not differ in DFA1 values (Tables S3–S5).

**Figure 1:**
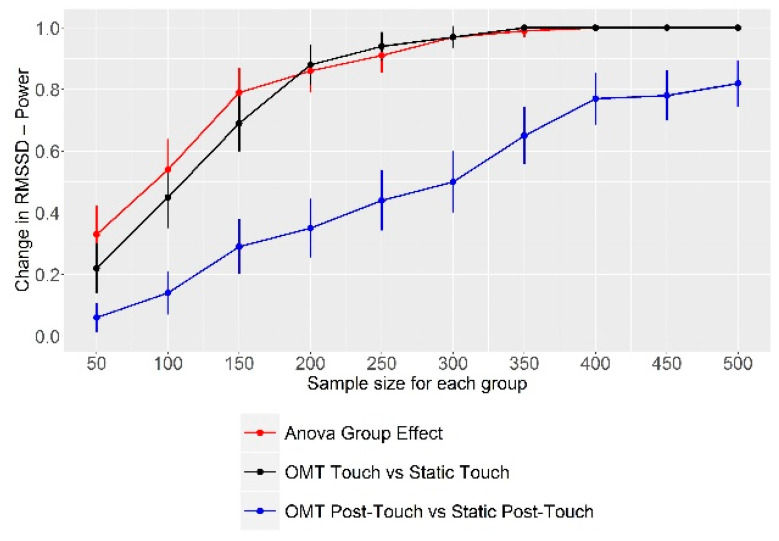
Power analysis via Monte Carlo simulation. The x-axis shows the sample size for each group: therefore, the total sample size for each point equals two times its corresponding x-axis value (e.g., a value of 100 on the x-axis corresponds to a total sample size of 200). For every sample size evaluated, the graph reports the power β for detecting a statistically significant change in RMSSD value: as revealed by the ANOVA group main effect; between the Dynamic Touch group and the Static Touch group during the intervention; between the Dynamic Touch group and the Static Touch group after the intervention. Abbreviations: RMSSD, root mean squared successive differences.

Regarding the Monte Carlo simulation, Figure 1 and Table S6 clearly show that our study was underpowered: indeed, to find a statistically significant difference in RMSSD values between the OMT Touch and Static Touch groups with a power of about 0.8, we would need a sample size of about 150 subjects per group (300 subjects in total). Furthermore, this sample size would increase for finding a difference in both the Touch and Post-Touch periods, respectively, about 175 subjects per group (350 in total), and about 500 subjects per group (1000 in total).

## 4. Discussion

The present paper aimed to assess whether OMT could induce changes in HRV metrics in preterm infants recovered in NICU, as HRV fluctuations can reveal both the ability of the organism to self-regulate and adapt to stressors and the state of newborn’s clinical conditions.

Regarding the primary outcome, i.e., RMSSD, the study failed in finding statistically significant changes between and within the two groups (OMT and Static Touch). Nonetheless, some interesting results were found. In particular, the two composite metrics aimed to specifically assess PNS and SNS modulation changed differently based on the kind of touch received by newborns: indeed, compared to static touch, OMT induced a parasympathetic modulation as revealed by an increase in the PNS index and a decrease in the SNS index both during and after the intervention. Moreover, concerning the within-group analysis, OMT elicited a parasympathetic modulation during the intervention compared to the Baseline, whereas static touch induced a particularly strong sympathetic activation, especially after the intervention had ended.

Regarding the frequency-domain metrics, the decrease in LF power and the lack of HF power change could point to a more balanced sympathovagal regulation [51]. On the other hand, since LF are influenced by baroreceptors activity [51], the lower LF power values seen during OMT could be due to a decrease in blood pressure mediated by the parasympathetic activation (as revealed by the increase in the PNS index). However, we have to remember that the actual threshold used for discriminating the different frequency bands derived from animal studies and that they were never validated in humans [64,65].

As another useful intervention for improving the conditions and development of preterm newborns is skin-to-skin care, it is interesting to compare the present findings with the effect of maternal contact on HRV. On the one hand, higher frequency of maternal contact correlated with higher HF in newborns [66]; on the other hand, skin-to-skin care have been shown to reduce the likelihood of HR decelerations [67], which could be considered as a risk factor for sepsis when pathological [20], and to help newborns balancing the stress and pain response to harmful events, i.e., heel stick [68,69]. Moreover, skin-to-skin care was found to elicit a parasympathetic response, especially in newborns whose parasympathetic tone is already low, whereas it could have little or no parasympathetic effect in infants with a higher PNS tone [70].

Concerning our study, such results might be interesting for two reasons: first, the skin-to-skin reduction in HR decelerations correlated with a decrease in RMSSD, SDNN, LF, and HF, and an increase in LF/HF [67,71]. Although not statically significant, we found lower RMSSD and SDNN after osteopathic touch compared to the Baseline, finding that was not obtained in the Static Touch group and that could reflect a similar adjustment in HR. Although lower RMSSD and SDNN could be regarded as a negative results, since these metrics reflect vagal and global autonomic modulation and are correlated with better neurodevelopment [54,55,56], we have to remember that HRV is a complex phenomenon, and single metrics do not always show a precise and linear meaning. For instance, in case of severe unconjugated hyperbilirubinemia, RMSSD rises steeply while HR decreases [72].

This is the reason why, in recent years, several composite scores have been developed and machine learning algorithms are taking the lead in HRV analysis [12,20,60,73]. As the organism is a complex network that functions through non-linear interactions between its parts, single and more linear HRV metrics may fail to detect changes in ANS modulation, homeostatic regulation or global health. On the other hand, composite scores obtained through the combination of different metrics has been shown to perform much better in predicting potential threat events (e.g., sepsis) or the evolution of clinical conditions than single metrics, whether they be linear or non-linear [60,63,73,74].

This could be the reason why we found significant results through the PNS and SNS indexes, but not through RMSSD and SDNN. In fact, RMSSD is included in the computation carried out to obtain the PNS index together with a non-linear metric such as SD1 [52]. On the other hand, using non-linear metrics, we found only one statistically significant result: after the intervention, the ApEn values were slightly higher in the OMT group. Possibly, since ApEn has been shown to be influenced by nociceptive and stressful events [57,58,59], the osteopathic touch might have induced a calming effect, as hypothesized by authors that saw similarities between OMT and gentle touch [36].

The second reason why skin-to-skin results are interesting for the present paper is that our two groups showed a statistically significant difference in HR at Baseline, which is actually a serious limitation of our study since, as a consequence, our groups cannot be considered as homogeneous. Furthermore, as HR is included in the computation of both PNS index and SNS index, this difference could have strongly biased our statistically significant findings. Nonetheless, the OMT group showed a lower HR, that is, a possible higher parasympathetic tone at Baseline. Therefore, although this difference could have biased the change in the PNS index and other metrics, in light of the evidence regarding skin-to-skin care, we might argue that, compared to skin-to-skin contact, OMT has a greater potential of inducing a parasympathetic tone, even in those infants who already show a PNS predominance.

Obviously, this is not to say that the faulty randomization does not have consequences: indeed, the higher peak in the SNS index shown by the Static Touch group could have been due to the already higher sympathetic tone of those infants. However, since the osteopathic touch induced a strong decrease in this index despite the lower HR at Baseline, only future studies with bigger samples and more accurate randomization could exactly point out how the heterogeneity between our groups have affected our findings.

It is noteworthy that the Monte Carlo simulation we performed showed that the analyzed sample was too low to effectively catch significant differences in the primary outcome (RMSSD) between the two groups. In fact, we would have needed a sample at least double in size compared to the one used. Considering the other findings, it is highly probable that we would need a higher sample size for detecting significant differences even in the other measured outcomes.

Interestingly, some findings were retained after the end of the intervention (e.g., the changes in the PNS and SNS indexes), whereas others became apparent only after the intervention (e.g., the changes in ApEn). These results can have a twofold meaning. First, OMT might indeed affect ANS regulation in a permanent manner, thus enabling preterm infants to better regulate their clinical conditions. In fact, OMT seems to have an initial metabolic effect in infants, which is followed by the autonomic regulation effect only after that [7]. Since C-tactile fibers are strictly connected with cerebral areas involved in the CAN and interoceptive network—the neurobiological network whose purpose is monitoring and regulating the internal milieu to maintain homeostasis and allow allostatic adaptation to stressors—eliciting such network could actually have long-lasting effects on homeostatic regulation and organism adaptation [24,36,75,76]. It is noteworthy that, compared to applying “mere” static touch, operators who focus on the sensations (e.g., tissue texture, temperature) they feel through touch, as osteopaths should do in their practice, can easily induce an activation of the interoceptive network in their patients [77].

As the second meaning, it could be useful to record whether the effects of OMT, or any other intervention, could last beyond the 5 min post-touch period assessed in the present study. Surely, due to the complexity of the clinical conditions of infants hospitalized in NICU and the many procedures that are administered to them, such long-term recording might prove difficult, both in actually doing and in interpreting. Nonetheless, it could be a source of useful data to understand the effects that interventions such OMT could have on infants’ autonomic regulatory capacities. Indeed, since NICU professionals touch infants hundreds of times per day to effectively take care of them [78], a manual intervention able to regulate the infants’ physiology and behavior could really improve neonatal and infant healthcare.

Concerning the precise effects of touch on infants, it is crucial to emphasize that there is a dearth of data regarding the effects of static touch and other types of touch on the baby’s state, i.e., quiet sleep, active sleep, or wakefulness. Although the literature indicates that these states involve varying degrees of autonomic functioning, as indicated by differences in HRV metrics [45], it is unknown whether there is a more optimal time to apply a particular type of touch. Thus, it is possible that using static touch or OMT during quiet sleep, as we did in this study, was not the best choice: it may have resulted in interruption of the infant’s sleep, which is known to be necessary for the infant’s health. Thus, future research should shed light on the optimal timing of intervention for various types of touch in neonatology to improve both the conduct of trials and the management of infants.

Lastly, we could argue that the present study added to the existing literature the fact that OMT indeed has an influence on the PNS, but more complex and accurate measurements are needed to highlight this effect. Despite the important limitations of the present study, in particular randomization and sample size, the analyzed data and the considerations put forth could help future research in defining more sound studies to detect the effects of OMT, or touch in general, on HRV metrics.

Should OMT actually induce a parasympathetic modulation on HRV, or a more global HRV rebalancing, it could be speculated that OMT could positively affect CAP activation [10,79]. Although usually the CAP is correlated mainly with vagal activity, thus making it a parasympathetic phenomenon, it is actually a response orchestrated by the CAN and performed by the ANS as a whole, with the PNS acting both as an afferent and an efferent way and the SNS acting as an efferent pathway [80].

## 5. Conclusions

The current study failed to demonstrate a change in RMSSD in preterm infants due to osteopathic touch. The other analyses showed, instead, that the approach used within the OMT was associated with increased PNS index and a decrease in SNS metrics. These findings suggest that a single osteopathic intervention might benefit autonomic effects during the preterm period, but it is necessary to perform more accurate studies with bigger samples, especially since positive results would provide important new insights into optimizing modern perinatal care approaches.

## Figures and Tables

**Table 1:** Extracted HRV metrics [12,13,51,52,53].

Parameter	Unit	Definition
HR	bpm	Number of heart beats per minute
RRI	n.	RR intervals obtained through the formula 60,000/HR
**Time-domain**		
SDNN	ms	Standard deviation of NN intervals
RMSSD	ms	Root mean square of consecutive RR interval differences
**Frequency-domain**		
LF Power	%	Relative power of the low-frequency band (0.04–0.2 Hz for newborns and 0.04–0.15 Hz for infants), obtained through the division of the absolute LF power by the summed absolute power of the LF and HF bands
HF Power	%	Relative power of the high-frequency band (0.20–2.00 Hz for newborns and 0.20–1.40 Hz for infants), obtained through the division of the absolute HF power by the summed absolute power of the LF and HF bands
**Non-linear**		
ApEn		Approximate entropy, which measures the regularity and complexity of a time series (mathematically speaking, ApEn is the negative natural logarithm of the conditional probability that a dataset of length N, having repeated itself for m samples within a tolerance r, will repeat itself again for one extra sample)
SampEn		Sample entropy, which measures the regularity and complexity of a time series (mathematically speaking, SampEN is obtained in the same way as ApEn, but excludes the counts where a vector is compared with itself)
DFA1		Detrended fluctuation analysis, which extracts the correlations between successive RR intervals over different time scales. Specifically DFA1 describes short-term fluctuations
**Composite**		
PNS index		An index regarding the parasympathetic nervous system modulation computed by the software Kubios through the analysis of the following metrics: mean RRI (longer RRI is tied to higher vagal modulation), RMSSD (it reflects vagal modulation on HR), and Poincaré plot index SD1 in normalized units (a non-linear metric tied to RMSSD)
SNS index		An index regarding the sympathetic nervous system modulation computed by the software Kubios through the analysis of the following metrics: mean RRI (shorter RRI is tied to higher sympathetic modulation), Baevsky’s stress index (a geometric parameter reflecting cardiovascular stress), and Poincaré plot index SD2 in normalized units (a non-linear metric tied to SDNN)

**Table 2:** General characteristics of the study sample at Baseline.

Characteristic	OMT (N = 50)	Static Touch (N = 46)	*p*-Value
Gestational age (weeks)	32.9 ± 4.4	33.9 ± 4.2	0.27
Birthweight (grams)	1967 ± 910	2173 ± 948	0.28
Sex	20 (40)	21 (46)	0.72
Heart rate	139.8 ± 16.3	145.6 ± 12.4	<0.001

Values shown are mean ± SD, except sex expressed as N (%). *p* values are from *t*-tests, and sex whose *p*-value is from χ^2^. Legend: OMT, osteopathic manipulative treatment.

**Table 3:** Tukey post hoc tests regarding RMSSD.

Group Comparison	RMSSD Difference (95% CI)	*p*-Value
Static T0–OMT T0	−0.813 (−2.033, 0.407)	0.394
Static T1–OMT T1	−0.724 (−1.944, 0.496)	0.529
Static T2–OMT T2	−0.433 (−1.658, 0.791)	0.911
Static T1–Static T0	0.313 (−0.547, 1.172)	0.902
Static T2–Static T1	−0.297 (−1.163, 0.569)	0.922
Static T2–Static T0	0.016 (−0.850, 0.882)	1.000
OMT T1–OMT T0	0.223 (−0.601, 1.048)	0.971
OMT T2–OMT T1	−0.587 (−1.412, 0.237)	0.318
OMT T2–OMT T0	−0.364 (−1.188, 0.461)	0.801

Legend: OMT, osteopathic manipulative treatment; RMSSD, root mean square of consecutive RR interval differences; static, static touch; T0, baseline; T1, touch period; T2, post-touch period.

**Table 4:** Tukey post hoc tests regarding SDNN.

Group Comparison	SDNN Difference (95% CI)	*p*-Value
Static T0–OMT T0	−1.746 (−5.310, 1.818)	0.722
Static T1–OMT T1	−0.636 (−4.201, 2.928)	0.996
Static T2–OMT T2	0.577 (−3.001, 4.154)	0.997
Static T1–Static T0	1.471 (−1.083, 4.026)	0.563
Static T2–Static T1	−0.465 (−3.038, 2.109)	0.995
Static T2–Static T0	1.007 (−1.567, 3.581)	0.871
OMT T1–OMT T0	0.362 (−2.089, 2.812)	0.998
OMT T2–OMT T1	−1.677 (−4.128, 0.773)	0.365
OMT T2–OMT T0	−1.316 (−3.766, 1.135)	0.637

Legend: OMT, osteopathic manipulative treatment; SDNN, standard deviation of NN intervals; static, static touch; T0, baseline; T1, touch period; T2, post-touch period.

**Table 5:** Tukey post hoc tests regarding PNS index.

Group Comparison	PNS Index Difference (95% CI)	*p*-Value
Static T0–OMT T0	−0.199 (−0.422, 0.024)	0.110
Static T1–OMT T1	−0.864 (−1.088, −0.641)	<0.001
Static T2–OMT T2	−0.894 (−1.118, −0.670)	<0.001
Static T1–Static T0	−0.310 (−0.439, −0.181)	<0.001
Static T2–Static T1	−0.007 (−0.137, 0.124)	1.000
Static T2–Static T0	−0.317 (−0.447, −0.186)	<0.001
OMT T1–OMT T0	0.355 (0.231, 0.479)	<0.001
OMT T2–OMT T1	0.023 (−0.101, 0.147)	0.995
OMT T2–OMT T0	0.378 (0.254, 0.502)	<0.001

Legend: OMT, osteopathic manipulative treatment; PNS, parasympathetic nervous system; static, static touch; T0, baseline; T1, touch period; T2, post-touch period.

**Table 6:** Tukey post hoc tests regarding SNS index.

Group Comparison	SNS Index Difference (95% CI)	*p*-Value
Static T0–OMT T0	0.487 (−1.814, 2.789)	0.990
Static T1–OMT T1	5.250 (2.948, 7.551)	<0.001
Static T2–OMT T2	6.675 (4.364, 8.986)	<0.001
Static T1–Static T0	0.952 (−0.766, 2.670)	0.606
Static T2–Static T1	2.796 (1.066, 4.526)	<0.001
Static T2–Static T0	3.748 (2.017, 5.478)	<0.001
OMT T1–OMT T0	−3.810 (−5.458, −2.163)	<0.001
OMT T2–OMT T1	1.371 (−0.277, 3.018)	0.165
OMT T2–OMT T0	−2.440 (−4.087, −0.792)	<0.001

Legend: OMT, osteopathic manipulative treatment; SNS, sympathetic nervous system; static, static touch; T0, baseline; T1, touch period; T2, post-touch period.

## Data Availability

Not applicable.

## Supplementary Materials

The following supporting information can be downloaded at: https://www.mdpi.com/article/10.3390/healthcare10050813/s1, Table S1. Tukey post hoc tests regarding LF relative power (%) computed through FFT. Table S2. Tukey post hoc tests regarding HF relative power (%) computed through FFT. Table S3. Tukey post hoc tests regarding ApEn. Table S4. Tukey post hoc tests regarding SampEn. Table S5. Tukey post hoc tests regarding DFA1. Table S6: Power analysis through Monte Carlo simulation regarding the primary outcome RMSSD. The power was calculated for the effect of the group variable in the ANOVA, and for the differences between OMT and Static Touch in the Touch and Post-Touch periods in the Tukey post hoc tests.
