# Supplementary material for: Osteopathic Manipulative Treatment Regulates Autonomic Markers in Preterm Infants: A Randomized Clinical Trial
Source: Healthcare (Basel). 2022 Apr 27;10(5):813. doi: 10.3390/healthcare10050813 (PMC9141319; doi:10.3390/healthcare10050813)
Supplement: Supplementary file 1 [file healthcare-10-00813-s001.zip › healthcare-1645482-supplementary.pdf]

Supplementary materials

Table S1. Tukey post-hoc tests regarding LF relative power (%) computed through FFT

Group Comparison	LF power difference (95% IC)	P-value
Static T0 – OMT T0	2.763 (-4.468, 9.993)	0.885
Static T1 – OMT T1	7.163 (-0.067, 14.394)	0.054
Static T2 – OMT T2	2.050 (-5.221, 9.320)	0.967
Static T1 – Static T0	3.787 (-2.960, 10.533)	0.597
Static T2 – Static T1	-2.798 (-9.587, 3.991)	0.848
Static T2 – Static T0	0.989 (-5.800, 7.778)	0.998
OMT T1 – OMT T0	-0.614 (-7.085, 5.857)	1.000
OMT T2 – OMT T1	2.316 (-4.155, 8.787)	0.911
OMT T2 – OMT T0	1.702 (-4.769, 8.173)	0.975

Legend: FFT, Fast-Fourier transformation; LF, low frequency band of heart rate variability; OMT, osteopathic manipulative treatment; Static, static touch; T0, baseline; T1, touch period; T2, post-touch period.

Table S2. Tukey post-hoc tests regarding HF relative power (%) computed through FFT

Group Comparison	HF difference (95% IC)	P-value
Static T0 – OMT T0	0.051 (-2.156, 2.257)	1.000
Static T1 – OMT T1	0.882 (-1.325, 3.089)	0.862
Static T2 – OMT T2	0.619 (-1.598, 2.836)	0.968
Static T1 – Static T0	1.228 (-0.621, 3.077)	0.403
Static T2 – Static T1	-0.153 (-2.014, 1.709)	1.000
Static T2 – Static T0	1.076 (-0.786, 2.937)	0.563
OMT T1 – OMT T0	0.397 (-1.377, 2.170)	0.988
OMT T2 – OMT T1	0.110 (-1.663, 1.884)	1.000
OMT T2 – OMT T0	0.507 (-1.266, 2.280)	0.964

Legend: FFT, Fast-Fourier transformation; HF, high frequency of heart rate variability; OMT, osteopathic manipulative treatment; Static, static touch; T0, baseline; T1, touch period; T2, post-touch period.

Table S3. Tukey post-hoc tests regarding ApEn

Group Comparison	ApEn difference (95% IC)	P-value
Static T0 – OMT T0	0.006 (-0.067, 0.079)	1.000
Static T1 – OMT T1	-0.040 (-0.113, 0.033)	0.617
Static T2 – OMT T2	-0.078 (-0.151, -0.004)	0.032
Static T1 – Static T0	-0.015 (-0.076, 0.045)	0.979
Static T2 – Static T1	-0.029 (-0.090, 0.032)	0.745
Static T2 – Static T0	-0.045 (-0.106, 0.017)	0.295
OMT T1 – OMT T0	0.030 (-0.028, 0.089)	0.668
OMT T2 – OMT T1	0.008 (-0.050, 0.066)	0.999
OMT T2 – OMT T0	0.039 (-0.020, 0.097)	0.407

Legend: ApEn, approximate entropy; OMT, osteopathic manipulative treatment; Static, static touch; T0, baseline; T1, touch period; T2, post-touch period.

Table S4. Tukey post-hoc tests regarding SampEn

Group Comparison	SampEn difference (95% IC)	P-value
Static T0 – OMT T0	-0.007 (-0.094, 0.080)	1.000
Static T1 – OMT T1	-0.020 (-0.107, 0.067)	0.986
Static T2 – OMT T2	-0.058 (-0.145, 0.029)	0.397
Static T1 – Static T0	-0.023 (-0.093, 0.046)	0.931
Static T2 – Static T1	-0.010 (-0.080, 0.060)	0.999
Static T2 – Static T0	-0.033 (-0.103, 0.037)	0.755
OMT T1 – OMT T0	-0.010 (-0.077, 0.057)	0.998
OMT T2 – OMT T1	0.028 (-0.039, 0.095)	0.833
OMT T2 – OMT T0	0.018 (-0.049, 0.085)	0.971

Legend: OMT, osteopathic manipulative treatment; SampEn, Sample entropy; Static, static touch; T0, baseline; T1, touch period; T2, post-touch period.

Table S5. Tukey post-hoc tests regarding DFA1

Group Comparison	DFA1 difference (95% IC)	P-value
Static T0 – OMT T0	-0.012 (-0.123, 0.098)	1.000
Static T1 – OMT T1	0.036 (-0.075, 0.146)	0.943
Static T2 – OMT T2	0.083 (-0.028, 0.195)	0.272
Static T1 – Static T0	0.028 (-0.084, 0.139)	0.981
Static T2 – Static T1	0.014 (-0.098, 0.126)	0.999
Static T2 – Static T0	0.042 (-0.070, 0.153)	0.897
OMT T1 – OMT T0	-0.020 (-0.127, 0.086)	0.994
OMT T2 – OMT T1	-0.034 (-0.140, 0.073)	0.947
OMT T2 – OMT T0	-0.054 (-0.161, 0.053)	0.701

Legend: DFA1, detrended fluctuation analysis 1; OMT, osteopathic manipulative treatment; Static, static touch; T0, baseline; T1, touch period; T2, post-touch period.

Table S6. Power analysis through Monte Carlo simulation regarding the primary outcome RMSSD. The power β was calculated for the effect of the group variable in the ANOVA, and for the differences between OMT and Static Touch in the Touch and Post-Touch periods in the Tukey post-hoc tests

N per group	ANOVA Group effect β	OMT T1 – Static T1 β	OMT T2 – Static T2 β
50	0.33	0.22	0.06
100	0.54	0.45	0.14
150	0.79	0.69	0.29
200	0.86	0.88	0.35
250	0.91	0.94	0.44
300	0.97	0.97	0.50
350	0.99	1.00	0.65
400	1.00	1.00	0.77
450	1.00	1.00	0.78
500	1.00	1.00	0.82

Legend: ANOVA, analysis of variance; OMT, osteopathic manipulative treatment; RMSSD, root mean square of consecutive RR interval differences; Static, static touch; T1, touch period; T2, post-touch period.
